# Effects of rye inclusion in dog food on fecal microbiota and short-chain fatty acids

**DOI:** 10.1186/s12917-023-03623-2

**Published:** 2023-05-10

**Authors:** Hanna Palmqvist, Sara Ringmark, Katja Höglund, Erik Pelve, Torbjörn Lundh, Johan Dicksved

**Affiliations:** 1 Department of Animal Nutrition and Management, Faculty of Veterinary Medicine and Animal Science, Swedish University of Agricultural Sciences, Uppsala, Sweden; 2 Department of Anatomy, Physiology and Biochemistry, Faculty of Veterinary Medicine and Animal Science, Swedish University of Agricultural Sciences, Uppsala, Sweden

**Keywords:** Arabinoxylan, Canine, Diet, Fiber, *Prevotella*

## Abstract

**Background:**

Rye intake has been associated with beneficial effects on health in human interventions, possibly due to dietary fiber in rye. In dogs, few studies have explored the effects on health of dietary fiber in general, and rye fiber in particular. The aim of this study was to investigate how inclusion of rye, compared with wheat, influenced fecal microbiota composition, short chain fatty acids (SCFA) and apparent total tract digestibility (ATTD) in dogs. Six male Beagle dogs (mean age 4.6 years, SEM 0.95 years; mean body weight 14.6 kg, SEM 0.32 kg) were fed three experimental diets, each for 21 days, including an adaptation period of six days and with 2–2.5 months between diet periods. The diets were similar regarding energy and protein, but had different carbohydrate sources (refined wheat (W), whole grain rye (R), or an equal mixture of both (RW)) comprising 50% of total weight on a dry matter (DM) basis. The diets were baked and titanium dioxide was added for ATTD determination. Fecal samples were collected before and in the end of each experimental period. Fecal microbiota was analyzed by sequencing 16S rRNA gene amplicons and fecal SCFA by high-performance liquid chromatography. Crude protein, crude fat, neutral detergent fiber, and gross energy (GE) in food and feces were analyzed and ATTD of each was determined. Univariate and multivariate statistical methods were applied in data evaluation.

**Results:**

Faecal microbiota composition, differed depending on diet (*P* = 0.002), with samples collected after consumption of the R diet differing from baseline. This was primarily because of a shift in proportion of *Prevotella*, which increased significantly after consumption of the R diet (*P* < 0.001). No significant differences were found for SCFA, but there was a tendency (*P* < 0.06) for higher molar proportions of acetic acid following consumption of the R diet. The ATTD of crude protein, crude fat, neutral detergent fiber, and GE was lower after consumption of the R diet compared with the other diets (*P* < 0.05).

**Conclusions:**

Consumption of the R diet, but not RW or W diets, was associated with specific shifts in microbial community composition and function, but also with lower ATTD.

**Supplementary Information:**

The online version contains supplementary material available at 10.1186/s12917-023-03623-2.

## Background

The composition of gut microbiota and its metabolic activities have significant impacts on the health of the host [1]. The gut microbiota is essential for several metabolic functions, such as degradation of dietary fiber, production of vitamins, and biotransformation of bile acids. It is also important for development of the immune system and in preventing colonization by pathogenic bacteria, among many other vital functions [2]. Specific members of the microbiota are known to be key species in this host-microbe interface, e.g., fiber-fermenting bacterial species such as members of the *Ruminococcaceae* family and *Prevotella* have been associated with healthy intestinal and metabolic status in both dogs and humans [3–6].

Dietary fiber escapes enzymatic digestion and absorption in the small intestine, and thus reaches the large intestine relatively unchanged. Dietary fiber includes both soluble and insoluble fiber [7]. Soluble fiber, e.g., arabinoxylan, β-glucan, and fructans, is readily fermented by the gut microbiota and is currently attracting attention due to its ability to create a healthy intestinal environment and metabolic homeostasis, and to its satiating effects [8]. The mechanisms behind these effects are not fully understood, but several factors seem to be involved. One factor is production of short-chain fatty acids (SCFA), primarily acetate, propionate and butyrate, as specific metabolites during bacterial fermentation [9]. Butyrate is the preferred energy source of colonocytes and is vital for their barrier function [10]. Moreover, SCFA lower the pH in the colon, favoring potentially beneficial bacteria such as *Bifidobacterium* and *Lactobacillus* [11, 12], while also restricting proliferation of bacteria associated with gastrointestinal disease in dogs, such as *Clostridium perfringens* and *Escherichia coli* [3, 4]. SCFA have been shown to increase satiety, both directly through central appetite regulation [13] and indirectly by stimulating release of satiety hormones [14, 15]. The species composition of microbiota is a key determinant for the type and levels of SCFA produced during fermentation of dietary fiber, but SCFA production is also dependent on the type and solubility of dietary fiber [16].

Whole grain cereals are rich in dietary fiber. In particular, rye, a cereal crop widely grown in Scandinavia, has a high content of dietary fiber, with arabinoxylan constituting the major part [17]. Studies on humans and pigs have shown effects of whole grain dietary fiber on microbiota, SCFA, and host metabolism, and associated health benefits [18, 19]. Whole grain rye products in particular are reported to be associated with these effects [19–22].

Some studies in dogs have investigated effects on the gut microbiota of consumption of high-carbohydrate or high-protein diets [23, 24], but these studies have mainly focused on the dietary starch content. Other studies have investigated the effects on microbiota and fermentation profile of specific fiber types, often included as dietary supplements [25, 26]. However, few studies have investigated the effects of whole grain in general on canine gut microbiota and their fermentation characteristics and, to our knowledge, only one published study has used rye as a carbohydrate source [27]. Given the interesting effects on gut microbiota and metabolism reported after rye inclusion in human interventions, it is relevant to study the effects of rye inclusion in dog food.

The aim of the present study was to investigate the effects of inclusion of rye, compared with wheat, in dog food on fecal microbiota, production of SCFA, and apparent total tract digestibility (ATTD).

## Methods

### Animals and housing

Six male purpose-bred Beagle dogs aged 1 to 7 years (mean 4.6 years, SEM 0.95 year) were subjected to three experimental diet periods with three different carbohydrate sources: wheat (W), mixed rye/wheat (RW), and rye (R). Mean body weight at the start of the study was 14.6 kg (SEM 0.32 kg, total range 13.6–15.7 kg) and mean body condition score (BCS) on a 9-point scale [28] was 5.3 (SEM 0.21, total range 5–6). Body weight and BCS were recorded once per week during the diet periods. The dogs were housed according to their regular routines, in indoor pens during evening and night and in outdoor pens, on gravel, in daytime. They were divided into one group of four and one group of two dogs, in accordance with their normal living conditions. Mean daytime outdoor temperature in the three periods was: Diet W: + 4.4 (range -4.4 to + 9.5) °C; diet RW: -4.0 (range -14.3 to + 2.1) °C; and diet R: + 18.5 (range + 5.8 to + 27.3) °C. Before the experiment started and between diet periods, the dogs were fed a standard commercial dog food with the following nutrient content on a dry matter (DM) basis: protein 23.1%, fat 16.2%, crude fiber 1.7%, and ash 5.1% (Science Plan, Medium, Adult, Advanced Fitness; Hills Pet Nutrition Inc., Topeka, KS, USA). Metabolizable energy (ME) content was 15.6 MJ/kg of food.

No signs of disease were detected in routine hematological and biochemical blood analyses (alanine aminotransferase, albumin, protein, creatinine, C-reactive protein) performed before the first diet period. Before each diet period, all dogs underwent physical examination [29] by the same veterinarian and were assessed as healthy. None of the dogs had been treated with any antimicrobial drugs during the six months preceding the study. All dogs were dewormed with Milbemax vet. (milbemycinoxim/prazikvantel) (Elanco, Stockholm, Sweden) prior to the experiment.

### Study design

**Fig. 1:**
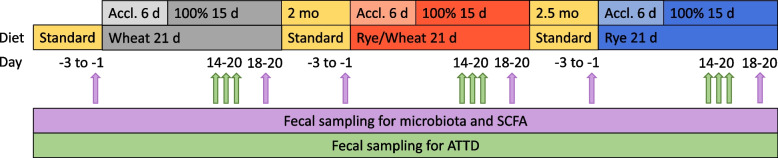
Flowchart of the study design. All dogs ate the three diets in the same order, with a washout period of their standard food in between. Three to one days before each diet period, one fecal sample per dog was taken to establish a baseline value of microbiota and short-chain fatty acids (SCFA) for that specific period. In the last week of each diet period fecal samples were collected for analysis of microbiota, SCFA and apparent total tract digestibility (ATTD). Accl = acclimatization period

Because the dogs were housed in groups and were known to consume feces occasionally, a cross-over study design was not possible. Thus it was decided to subject all dogs to the three different diets in the same order. An outline of the study design is shown in Fig. 1. The W diet was fed in the first period and the order of the two other diets was randomized to RW followed by R. Each diet period started with a six-day acclimatization period, in which the experimental diet was mixed with the dogs’ standard food in increasing amounts. The experimental diet was then fed to the dogs at 100% for 15 days. The diet periods were separated by 2–2.5 months, during which the dogs were fed their standard commercial food as described above.

### Diets and feeding

The experimental diets (ingredient list in Table 1, composition in Table 2) were designed to be as similar as possible in terms of energy and protein, but with differences in type of carbohydrates. Modified Atwater factors (protein 14.6 kJ/g, fat 35.6 kJ/g, nitrogen free extract 14.6 kJ/g) [7] were used to estimate the ME of the diet ingredients before formulation. Portion size was calculated based on this estimation. In all diets, the carbohydrate source consisted of food-grade flour (Kungsörnen, Sweden). The W diet contained refined wheat flour, the RW diet a mixture of coarsely ground whole grain rye meal and refined wheat flour, and the R diet rye meal alone. The flours were added in the respective diets to comprise 50% of weight (DM basis). In the mixed (RW) diet, the two flours were added to comprise 25% each of DM. In all diets, the flour was mixed with minced beef (Bravo Hundfoder AB, Klippan, Sweden) and chicken (OY MUSH ltd, Jakobstad, Finland), water, and rapeseed oil (in the RW and R diets to balance the energy) in an industrial mixer (Elektro Helios, Stockholm, Sweden). For measurement of ATTD, the inert marker titanium dioxide (TiO_2_) was added in an amount of 2 g/kg food. The mixture was spread on an oven tray and baked for 25–30 min at 180 °C in a steam oven to a core temperature of 90–100 °C. The baked food was allowed to cool at room temperature for an hour. To compensate for water loss during cooking and cooling, the baked food was then weighed and cold water was added to make up for the difference in weight compared with before cooking. Finally, the food was mixed with fish oil, vitamin premix (WorkingDog Multivitamin, Trikem AB, Malmö, Sweden; for composition see Supplementary Table 1, Additional File 1), and minerals (Calphosum D, Aptus, Orion Pharma Animal Health, Danderyd, Sweden; for composition see Supplementary Table 2, Additional File 1), portion-packaged, and immediately frozen to -20 °C. Before feeding, the food was thawed in a refrigerator.

**Table 1:** List of ingredients used in the experimental diets (g/kg)

Ingredient	Wheat (Diet W)	Rye/Wheat (Diet RW)	Rye (Diet R)
Rye meal, whole grain	0	132	264
Wheat flour, white	269	135	0
Beef meat	213	196	182
Chicken meat	328	348	366
Fish oil	13	12	12
Rapeseed oil	0	4	6
Water	133	131	128
Titanium dioxide	2	2	2
Vitamin	21	20	20
Mineral	21	20	20

**Table 2:** Chemical composition (% of dry matter) and energy content (MJ/kg dry matter) of the experimental diets

Diet	Wheat (Diet W)	Rye/Wheat (Diet RW)	Rye (Diet R)
Dry matter, %	49.9	51.4	49.6
Crude protein, %	22.5	20.8	20.5
Crude fat, %	29.5	32.3	31.1
N-free^1^ extract, %	43.3	41.4	42.2
Crude fiber, %	0.58	0.89	1.24
Neutral detergent fiber, %	2.9	6.3	6.6
Ash, %	4.1	4.6	5
Gross energy, MJ/kg	24.7	24.6	24.5
Metabolizable energy^2^, MJ/kg	21.3	21.2	21.0
Metabolizable energy^2^, MJ /kg food	10.6	10.9	10.4

^1^Nitrogen-free extract (calculated)

^2^Metabolizable energy calculated in accordance with NRC[7]

Daily energy requirement for each dog was calculated according to National Research Council [7] recommendations for the average laboratory kennel dog, i.e., 543 kJ ME/kg metabolic body weight (BW^0.75^ kg), based on estimated ideal body weight of the dogs at the beginning of the first diet period. The food was divided into two meals per day, offered to the dogs at approximately 08.00 h and 16.00 h. Before feeding, the dogs were moved to individual indoor pens, where they were allowed to eat for 10–15 min and then returned to the kennel. Water was made available ad libitum. Since the capability of the dogs for digestion of rye was not known, an ethical exclusion limit was set at maximum 5% weight loss per week, or 10% during the complete diet period, or a two-point decrease in BCS over two weeks, along with a limit of three days of total food refusal. In order to avoid interference with dietary effects on gut microbiota or ATTD, the intention was to keep the portion size unchanged during the diet periods. However, for four dogs during the RW period and one dog during the R period, the portion size had to be increased in the last three days of the period to maintain body weight above exclusion level (by 25% for RW and by 10% for R).

### Fecal samples

Fecal samples for analysis of microbiota composition and SCFA were collected immediately after voiding, once at 1–3 days before the start of each period to get baseline values, and once during the last three days of each experimental period. At sampling, care was taken not to collect feces that had been in contact with the ground. Samples were stored at -80 °C until analysis. Fecal samples for determination of ATTD were collected once a day for three days in the middle of each diet period, except from one dog in the W period and one dog in the R period, where only two samples could be collected. These samples were kept at -20 °C until analysis. Fecal consistency was assessed on a scale from 1 to 5, where 1 was described as very loose (diarrhea), 5 was dry and hard, and 4 was assessed as optimal (fecal scoring system for dogs; Royal Canin SAS 2013, www.royalcanin.ca). Scoring was performed immediately after voiding, by either the first author or the animal keeper, during walks or in the morning when the dogs were released into their outdoor pen.

### Microbiota analysis

DNA was extracted using QIAamp Fast DNA Stool Mini Kit (Qiagen Gmbh, Hilden, Germany) according to the manufacturer’s protocol, but with the modification of using bead-beating to break down bacterial cell walls. The bead-beating step was carried out by adding 0.3 g sterilized 0.1 mm zirconia/silica beads to the samples and running them in a Precellys24 sample homogenizer (Bertin Technologies, Montigny-le-Bretonneux, France), 6500 rpm, for 2 × 1 min. The extracted DNA was kept at -20 °C until further analysis. PCR amplicons were generated from the V3-V4 region of the 16S rRNA gene using the primers F341 and R805 and with Phusion® High-Fidelity PCR chemistry (Thermo Fisher Scientific Inc., Waltham, MA, USA). The first PCR run was initiated with denaturation at 98 °C for 30 s, followed by 30 cycles with denaturation at 98 °C for 10 s, hybridization at 60 °C for 30 s, and elongation at 72 °C for 4 s, and the run was ended with a final elongation at 72 °C for 2 min. Amplicons were cleaned using Agencourt AMPure XP magnetic beads according to the manufacturer’s instructions (Beckman Coulter Inc., Bromma, Sweden). In the second PCR run, forward and reverse barcode primers were added to barcode each sample individually. The primers contained both barcode and Illumina adaptor sequences and the PCR amplicons were generated with Phusion® High-Fidelity Master Mix (Thermo Fisher Scientific Inc.). The conditions for the second PCR run were the same as for the first except for a 5 s elongation in each cycle and 10 cycles in total. Amplicons were then cleaned as after the first PCR. The samples were quantified using a Qubit® 3.0 Fluorometer (Invitrogen, Thermo Fisher Scientific Inc.), and samples were then pooled in equimolar amounts. Amplicons were sequenced on the MiSeq Illumina platform, using the v3 kit (2 × 300 bp) at the National Genomics infrastructure (NGI) hosted by SciLifeLab, Solna, Sweden.

The raw sequence dataset contained in average 84,397 (inter quartile range: 63,114 to 102,119) paired sequences/sample. The amplicon sequences were analyzed with the software Mothur v.1.41.0 [30]. Paired-end reads joined by the ‘make.contigs’ command were filtered by the ‘screen.seqs’ command to remove sequences deviating from the 90% majority of sequences with regard to overlap length and number of mismatches, as well as minimum and maximum length of the joined reads. In addition, sequences with homopolymers larger than eight nucleotides and sequences with ambiguous bases were removed. The sequences were aligned to the Silva database version 132 [31], and the aligned sequences were filtered to remove sequences deviating from the 90% majority with regard to start and end position of the aligned sequences compared with the reference. Classified sequences (cutoff value 80) were filtered to remove hits to chloroplasts, mitochondria, and eukaryotes, and unknown hits. The filtered dataset contained in average 54,676 (inter quartile range: 40,243 to 65,363) merged sequences/sample. Phylotypes were produced with the phylotype, make.shared, and classify.otu commands.

### Short-chain fatty acid analysis

Three SCFA (acetate, propionate, and butyrate) were analyzed in 0.5 g fecal matter dissolved in 1 mL 5 mM H_2_SO_4_, as previously described [32], using a high-performance liquid chromatography system consisting of an Alliance 2795 separation module and a 2414 RI Detector (Waters Corp. Milford, MA, USA). Column packet ReproGel H 9µ 300*8 mm was used as the separation column and a ReproGel H, 9µ 30*8 mm (Dr. A. Maisch, Ammerbuch, Germany) was used as a pre-column. Due to interference from ingested gravel in some fecal samples, the concentrations of the individual SCFA were converted to proportions before statistical analysis.

### Analysis of food and feces

The experimental food and fecal samples were dried in a freeze dryer for 72 h before further analysis. For DM determination, the samples were dried at 103 °C for 16 h, followed by cooling in a desiccator before weighing [33]. Ash was determined by incinerating the dried samples in an oven at 550 °C for three hours and then cooling in a desiccator before weighing. Total nitrogen was determined according to the Kjeldahl method [34], using a 2020 digester and a 2400 Kjelltec analyzer (FOSS Analytical A/S, Hilleröd, Denmark), and crude protein was calculated as N × 6.25. Crude fat was analyzed according to Commission Directive EC/152/2009 [35] using a Soxtec extraction unit (FOSS Analytical A/S, Hilleröd, Denmark). Crude fiber was analyzed by boiling a sample first in H_2_SO_4_ solution and then in KOH solution [36]. Neutral detergent fiber was analyzed as previously described [37]. Gross energy (GE) was measured on a Parr isoperobol Bomb Calorimeter 6300 (Parr Instrument Company, Moline, Illinois, USA). Titanium dioxide was analyzed according to Short et al*.* [38] and ATTD was calculated as: ATTD (%) = 100-[(% TiO_2_ in food/% TiO_2_ in feces) × (% Nutrient in feces/% Nutrient in food) × 100] [26].

### Statistical analyses

Principal coordinate analysis (PCoA) based on Bray Curtis distances and analysis of similarity (ANOSIM) was used to analyze microbial community structure and evaluate the effects of the dietary interventions on the microbiota. A similarity percentage test was used to identify taxa primarily responsible for differences between groups identified in ANOSIM analysis. To assess changes in relative abundances of microbial taxa due to the intervention, analysis of variance (ANOVA) was performed with a linear mixed effects model fitted with diet, time, and the interaction diet:time as fixed effects and dog as random effect. The time factor had two levels denoting if samples were collected before or after the diet period. The model for each taxon was checked with diagnostic plots for homoscedasticity and normality. If the model did not meet the criteria, but a model with natural logarithm-transformed data did, the latter was used instead. *Post-hoc* comparisons of estimated marginal means were only made between baseline and post-diet samples within each diet, or between different diets but at the same time point. These comparisons were made with Tukey’s adjustment. Only microbial taxa with a total mean relative abundance of minimum 1% were analyzed. A linear mixed effects model with a similar structure was used to test for differences between molar proportions of SCFA. For ATTD, verification of equal body weight and BCS at the start of each period, and fecal score and DM, a linear mixed effects model was fitted with diet as fixed effect and dog as random effect. The PCoA and ANOSIM analyses were carried out using the software Past [39] and the linear mixed effects models using the lmer-function in R [40]. Differences were regarded as significant at P ≤ 0.05.

## Results

All dogs but one completed all three diet periods. One dog lost more weight than the set limit of 5% in one week when fed the R diet, and was excluded from that period. All dogs but one ate all food provided during all periods. The dog that refused some food primarily did so in the morning and mostly during the R diet period. To compensate, that dog was offered up to 50% extra at the afternoon meal, but still ate 23% less during the period than the calculated requirement. Mean food consumption per kg body weight and day for the periods was: W 24.6 g (range 23.4–25.7 g), RW 26.3 g (range 24.6–27.7 g), and R 24.4 g (range 21.1–26.1 g). Mean weight loss per diet period ranged from 1.8% to 4.0% (values from excluded dog in R diet not included). Body weight and BCS at the start and end of each diet period are presented in Supplementary Table 3 and Supplementary Table 4, respectively, in Additional File 2. Body weight and BCS at the start of each diet period did not differ between periods. We discovered small amounts of gravel in some of the fecal samples, likely after ingestion in the outdoor pen.

### Microbiota

**Fig. 2:**
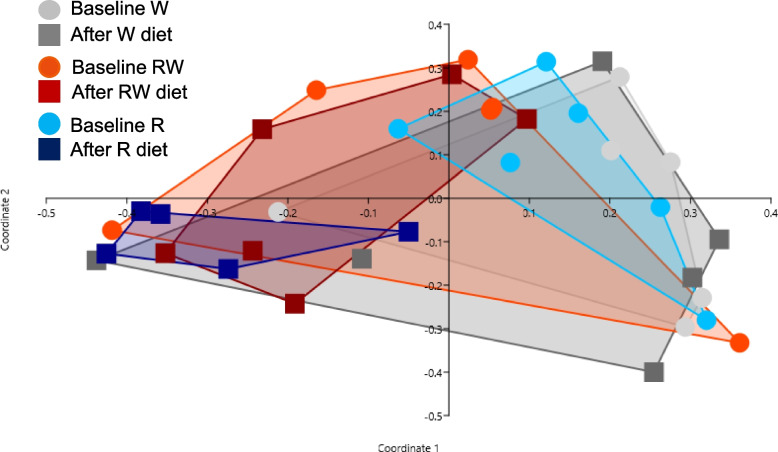
Principal coordinate analysis (PCoA) plot of fecal microbial composition before (baseline) and after each experimental diet. Baseline samples are represented by point symbols and samples collected after the diets are represented by filled squares. Diet periods are represented by different colors: grey = wheat diet (W); red = rye-wheat diet (RW); blue = rye diet (R)

**Fig. 3:**
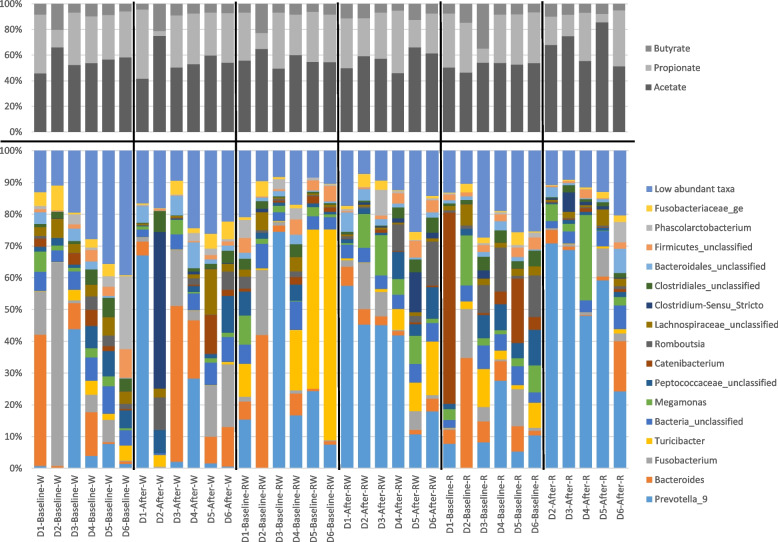
Barcharts showing relative proportions of short chain fatty acids (upper barchart) and microbiota (lower barchart) for individual samples. D1-D6 in sample legends represent the individual dogs whereas “baseline” and “after” indicate if it was before or after the diet period. For easier interpretation, only taxa with an average relative abundance > 2% are shown in the figure. All taxa with lower average relative abundance are shown as low abundant taxa. W = wheat diet, RW = diet with equal mixture of whole grain rye and refined wheat, R = whole grain rye diet

**Fig. 4:**
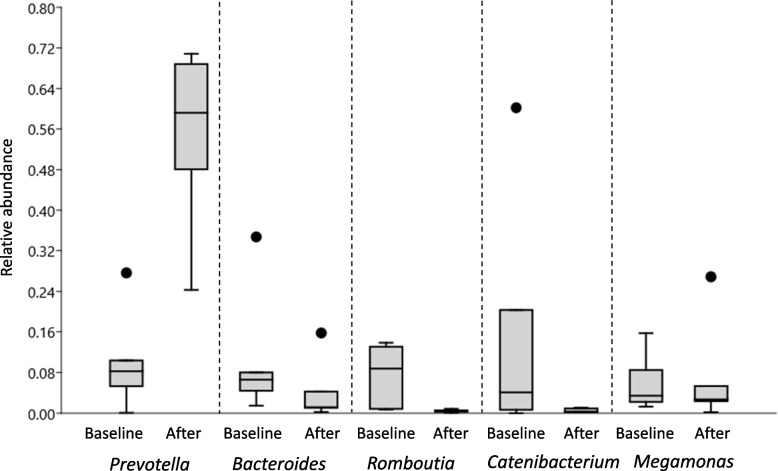
Box plots showing relative abundance, at baseline and after the R diet, of the top five taxa of importance for the difference seen in analysis of similarity (ANOSIM), according to similarity percentage tests. The box represents the 25–75 percentage quartiles and the horizontal line in the box represents the median value. Whiskers represent minimum and maximum values and dots represent outliers

Microbial composition in the baseline fecal samples did not differ significantly between the three periods according to PCoA and ANOSIM (Fig. 2). However, there was variation between individual dogs as well as temporal variation between individual samples within dogs (Fig. 3). Following both the R and RW diet periods, *Prevotella* was the overall most abundant genus in feces and clearly dominant (mean 54% and 36% of sequences, respectively). PCoA and ANOSIM revealed differences in microbial composition linked to diet (ANOSIM; *P* = 0.002, *R* = 0.19). There were no differences when comparing baselines and samples collected after the W and RW diet periods, but in dogs fed the R diet microbiota composition in feces differed from baseline (ANOSIM; *P* = 0.014, *R* = 0.67) (Fig. 2). Similarity percentage test revealed that the taxa of highest importance for the observed difference following the R diet were: *Prevotella*, *Catenibacterium*, *Bacteroides*, *Romboutsia*, and *Megamonas* (Fig. 4).

ANOVA was performed on 22 taxa detected in mean relative abundance > 1%. This represented in total 86% of the generated sequences. Fourteen of these taxa displayed differences in at least one effect in ANOVA (Table 3). Natural logarithm-transformed values were used for 16 taxa.

**Table 3:** Mean microbial relative abundance at baseline when the dogs were fed a commercial diet and after each three week experimental diet period

	Wheat (Diet W)		Rye/Wheat (Diet RW)		Rye (Diet R)		D^1^	T^2^	D:T
	Baseline	After	Baseline	After	Baseline	After			
*Prevotella*^*#*^	9.6 ± 6.9	16.6 ± 11.0	23.1 ± 10.8	36.4 ± 7.4	9.9 ± 3.8	54.3 ± 8.5	*	**	*
*Bacteroides*	10.9 ± 6.5	15.4 ± 7.2	9.7 ± 6.5	3.4 ± 0.8	10.2 ± 5.0	4.5 ± 2.9			
*Fusobacterium*^*#*^	15.3 ± 10.0	9.7 ± 3.7	4.1 ± 3.3	5.3 ± 2.2	5.6 ± 2.6	2.7 ± 1.6			
*Turicibacter*	2.4 ± 0.8	0.8 ± 0.6	24.5 ± 11.3	5.7 ± 2.7	4.4 ± 1.9	0.5 ± 0.3	**	**	
*Megamonas*	2.6 ± 0.8	1.5 ± 0.7	2.8 ± 1.3	6.5 ± 2.1	5.6 ± 2.3	7.5 ± 4.9			
Peptostreptococcaceae uncl	4.2 ± 1.2	3.8 ± 1.9	2.9 ± 1.1	4.3 ± 1.6	5.9 ± 1.5	1.0 ± 0.2		*	*
*Catenibacterium*	2.2 ± 0.8	2.5 ± 2.0	1.3 ± 0.4	0.4 ± 0.1	14.9 ± 9.6	0.6 ± 0.2		*	
*Romboutsia*^*#*^	1.7 ± 0.7	3.0 ± 1.7	1.2 ± 0.6	4.3 ± 2.3	6.4 ± 2.6	0.4 ± 0.1			*
Lachnospiraceae uncl	4.2 ± 0.6	3.7 ± 2.2	2.3 ± 0.9	0.8 ± 0.1	2.8 ± 0.8	2.1 ± 0.8	*	*	
*Clostridium *sensu stricto* 1*	0.1 ± 0.0	8.4 ± 8.2	0.2 ± 0.2	2.8 ± 2.0	0.5 ± 0.4	1.8 ± 1.1		***	
Clostridiales uncl	3.1 ± 0.9	2.2 ± 1.0	1.8 ± 0.7	2.3 ± 0.8	2.9 ± 0.7	1.2 ± 0.3			
Bacteroidales uncl^#^	1.8 ± 0.7	3.8 ± 1.3	1.4 ± 0.4	2.5 ± 0.8	1.4 ± 0.3	2.6 ± 1.4			
Firmicutes uncl	3.0 ± 1.3	0.7 ± 0.2	3.1 ± 0.9	2.4 ± 0.8	2.4 ± 0.4	1.2 ± 0.5		**	
*Phascolarctobacterium*	5.2 ± 3.6	0.9 ± 0.4	2.3 ± 0.9	1.7 ± 1.3	0.7 ± 0.2	1.3 ± 1.3	*	*	
*Fusobacteriaceae_ge*^*#*^	3.3 ± 1.1	2.8 ± 1.0	1.3 ± 0.7	1.9 ± 0.6	1.6 ± 0.6	1.0 ± 0.4			
*Peptoclostridium*^*#*^	2.7 ± 0.6	0.7 ± 0.3	1.4 ± 0.6	0.9 ± 0.3	2.8 ± 0.7	0.5 ± 0.1		***	
*Alloprevotella*	1.4 ± 0.7	2.9 ± 1.0	0.6 ± 0.2	1.6 ± 0.8	0.6 ± 0.3	1.4 ± 0.7			
Prevotellaceae uncl	0.7 ± 0.4	1.8 ± 0.9	1.0 ± 0.3	1.8 ± 0.6	0.5 ± 0.2	1.7 ± 0.6		*	
*Faecalibacterium*	3.5 ± 1.6	0.04 ± 0.0	0.6 ± 0.2	0.1 ± 0.0	2.4 ± 0.9	0.4 ± 0.2		***	
Clostridiaceae 1 uncl	0.1 ± 0.0	2.6 ± 2.4	0.3 ± 0.2	2.3 ± 1.3	0.6 ± 0.5	1.2 ± 0.6		**	
*Sutturella*	1.5 ± 0.3	0.8 ± 0.3	0.8 ± 0.2	0.8 ± 0.2	1.3 ± 0.2	1.0 ± 0.2			
*Anaerobiospirillum*	1.2 ± 0.6	1.3 ± 0.7	0.5 ± 0.2	0.3 ± 0.2	2.8 ± 2.1	0.03 ± 0.0		*	
*Prevotella*/*Bacteroides *ratio	3.4 ± 2.1	2.9 ± 2.5	13.9 ± 7.0	13.0 ± 3.9	2.5 ± 1.1	67.4 ± 35.8			
Firmicutes/Bacteroidetes ratio	9.5 ± 5.0	80.4 ± 73.3	2.6 ± 1.2	1.5 ± 0.7	3.4 ± 0.9	0.4 ± 0.1		*	

Data shown for taxa with total mean relative abundance > 1%. Values in % ± SEM. ^1,2^Significant difference in diet (D) and/or time (T) and/or the interaction (D:T) in analysis of variance (ANOVA) at: *P ≤ 0.05, **P ≤ 0.01, ***P ≤ 0.001. The time factor had two levels denoting if samples were collected before or after the diet period. #Non-logarithmic values were used in the model, (*uncl* = unclassified)

Relative abundance of *Prevotella* in feces increased numerically after all diet periods, but the difference was only significant after the R diet period (*P* < 0.001). Relative abundance was also higher following the R diet compared with the W diet (*P* < 0.007). Furthermore, diet had an effect from baseline to after diet, i.e., the interaction was significant, for *Romboutsia* and for an unclassified member of the family *Peptostreptococcaceae*, with abundance of both decreasing after the R diet period (*P* < 0.02 for both). For the other taxa with significant changes detected in ANOVA, differences were found for time and in some instances also for diet, but not for their interaction. *Prevotella*/*Bacteroides* ratio showed a tendency for differentiation depending on diet and time (*P* < 0.08 and *P* < 0.06, respectively), but not for their interaction. Firmicutes/Bacteroidetes ratio was different for time (*P* < 0.05), but the interaction effect was not significant.

### Short-chain fatty acids

**Fig. 5:**
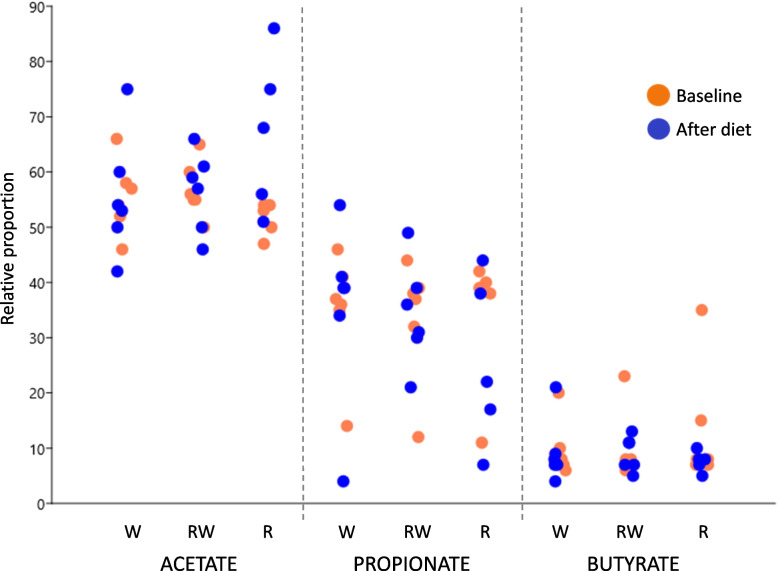
Jitter plot showing molar proportions of acetate, propionate, and butyrate as proportion of total short-chain fatty acids (SCFA). There were no differences between diets (*P* > 0.05)

The relative proportions of acetate, propionate, and butyrate in feces showed no overall differences for any of the diets (*P* > 0.05), but for acetic acid there was a tendency for a difference in time (*P* < 0.08) and for the interaction between time and diet (*P* < 0.06) (Fig. 3 and Fig. 5). *Post-hoc* comparisons of the marginal means with Tukey’s adjustment revealed higher molar proportions of acetic acid after the R diet (mean before 52.2% and SEM 1.4%, mean after 67.0% and SEM 6.3%; *P* = 0.005).

### Apparent total tract digestibility

Mean ATTD of crude protein, crude fat, neutral detergent fiber, and GE was lower in all cases for the R diet compared with the other two diets, while the RW diet only differed from the W diet in terms of lower ATTD for GE (Table 4).

**Table 4:** Emmeans of apparent total tract digestibility (ATTD), % ± standard error

	Wheat (Diet W)	Rye/Wheat (Diet RW)	Rye (Diet R)	*P-value*
Crude protein	89.3 ± 0.79^a^	87.0 ± 0.79^a^	79.6 ± 0.87^b^	< 0.001
Crude fat	96.8 ± 0.68^a^	97.0 ± 0.68^a^	95.7 ± 0.70^b^	0.01
Neutral detergent fiber	69.9 ± 3.62^a^	64.0 ± 3.62^a^	34.0 ± 3.92^b^	< 0.001
Gross energy	93.6 ± 0.66^a^	90.5 ± 0.66^b^	83.1 ± 0.74^c^	< 0.001

^abc^ATTD values within rows with different superscript letters differ significantly between the diets (*P* < 0.05)

### Fecal score and dry matter

Mean fecal score for the last seven days of each diet period did not differ between diets (W: mean 3.5, SEM 0.2, range 1–5; RW: mean 3.6, SEM 0.2, range 2.5–5; R: mean 3.1, SEM 0.2, range 2–4). Mean fecal DM also did not differ between diet periods (W: mean 32.1, SEM 5.6, range 20.4–41.8%, RW: mean 28.0, SEM 0.6, range 26.2–29.6%, R: mean 32.3, SEM 1.9, range 27.5–38.1%).

## Discussion

This study investigated how diets with different inclusion levels of whole grain rye influenced fecal microbiota composition, SCFA profile, and ATTD in dogs. The main differences were observed with the highest inclusion level of rye (50% of DM), which caused a change in microbial composition, mainly due to an increase in the relative abundance of *Prevotella*.

Multivariate analysis showed a difference in microbial composition after the R diet compared with the other diets. This difference was primarily driven by an increase in *Prevotella* and a decrease in *Catenibacterium*, *Bacteroides*, *Romboutsia*, and *Megamonas* (Fig. 4). Univariate analysis showed that the relative abundance of several taxa changed significantly between the baseline and after the diet periods in general. For *Prevotella*, *Romboutsia*, and an unclassified member of *Peptostreptococcaceae*, the model revealed that the changes were significant only for the R diet. The detection of higher relative abundance of *Prevotella* is in line with findings in a previous study on humans examining the effect of rye-kernel bread versus white wheat bread on gut microbiota [41]. That study found that *Prevotella* increased after rye bread compared with white wheat bread consumption, but that *Bacteroides* showed a tendency to decrease with rye bread consumption compared with wheat bread. In a study on pigs, increased abundance of *Prevotella* following consumption of an arabinoxylan-rich diet, although derived from wheat, has been reported [42]. A recently published study on Beagle dogs fed a vegetarian diet supplemented with feather meal and either corn meal, rye, or fermented rye did not find any significant influence on microbiota composition, but addition of rye, fermented or not, increased the proportion of the phylum Bacteroidetes, in particular in some dogs [27]. *Prevotella* is a dominant member of the Bacteroidetes phylum, and thus our results point in the same direction as those in that study. Our finding of increased relative abundance of *Prevotella* after the R diet is interesting, since a previous study in humans and mice found that high abundance of *Prevotella* had a favorable impact on glucose metabolism when the test subjects consumed a fiber-rich diet compared with a refined carbohydrate product [6]. It would be interesting to investigate, using a larger sample, whether this connection also exists in dogs.

Since the abundance of the different taxa was measured as relative proportions, it is plausible that the difference seen in PCoA and ANOSIM was driven by the large increase in *Prevotella* after the R diet and that the reductions in the other taxa were mainly a consequence of that. However, there was large variation in relative abundance of taxa, both between and within the dogs, which in combination with a small sample size probably masked other changes in microbial composition linked to the experimental diets.

Firmicutes/Bacteroidetes ratio and *Prevotella*/*Bacteroides* ratio have previously been shown to be affected by carbohydrate to protein ratio in the diet of humans [43, 44] and dogs [45]. In humans, the amount of complex carbohydrates is suggested to be the main influence [44]. Our study, although based on a limited number of animals, showed similar indications, with a lower Firmicutes/Bacteroidetes ratio after the R diet period and a tendency for higher *Prevotella/Bacteroides* ratio depending on diet and time. The change in *Prevotella* abundance was likely the main contributor to the changes in both ratios. The shift in ratio after the R diet period aligns well with the findings from the previous mentioned study [27], which also observed that inclusion of rye, but not corn, shifted the Firmicutes/Bacteroidetes ratio in favor of Bacteroidetes.

For the other taxa identified in this study, a few published papers have reported associations to dietary fiber. A decrease in *Catenibacterium* abundance was indicated as important for the difference in ANOSIM after the R diet, whereas a previous study in pigs observed an increase in *Catenibacterium* abundance after inclusion of oat bran in the diet [46]. *Megamonas* abundance has been found to increase in dogs fed a diet based on raw meat supplemented with inulin, compared with a control [47]. *Faecalibacterium*, which has previously been associated with fiber-rich diets [12, 48] and colonic health in dogs [3], decreased in relative abundance after all diets in our study.

The three experimental diets were designed to be as similar as possible in terms of fat content, but all experimental diets had a higher fat content than the dogs’ standard diet. This change from the normal diet might have contributed to some of the changes detected in microbiota composition, as a previous study on dogs found that consumption of a high-fat, low-starch diet led to a decrease in *Prevotella* compared with a high-starch, low-fat diet [49]. However, the high fat content in the diets in our study did not seem to have a negative effect on *Prevotella*.

Statistical analysis of fecal SCFA indicated a tendency for an increased proportion of acetic acid depending on diet, which was due to a difference between baseline R diet and post R diet, but no other differences were found. Acetate has been reported to suppress appetite in mice [13], indicating one possible way in which rye can promote satiety. *Prevotella-*dominant microbiota has been shown to correlate with increased relative production of propionate rather than acetate, although with some differences depending on substrate [16, 50]. However, high proportional acetate production has been seen in combination with *Prevotella*-dominated microbiota in finisher pigs fed pea fiber [51]. The total amount of SCFA produced is also reported to depend on the dominant genera. *Prevotella*-dominated fecal inoculum has been found to increase the amount of SCFA in vitro compared with *Bacteroides* fermenting the same type of fiber, indicating higher fiber-utilizing capacity in *Prevotella* [16]. We expected total fecal SCFA to increase with increased inclusion of whole grain rye, due to the larger quantity of fermentable fiber. However, the absolute levels of SCFA could not be determined, because some of the dogs apparently ingested small amounts of gravel from their outdoor pen, which contaminated the fecal samples.

In the R diet period, ATTD was lower for all nutrients, including GE, compared with the other diet periods. This is in line with findings in a study on pigs comparing whole grain cereals from rye and wheat [52], where lower digestibility (both ileal and total tract) was seen for rye and attributed, in part, to higher viscosity. A study on dogs in which rye was added at 20%, as-is, to a basic diet found no differences in ATTD of crude protein or crude fat [53]. Dog foods containing other cereals have been observed to vary in digestibility of nutrients depending on carbohydrate source [54], with digestibility decreasing with increasing fiber inclusion [55]. The difference in ATTD between diets in our study may also have been due to the rye meal being more coarsely ground than the wheat meal. Our fecal collection period of three days, with only one sample per day, might also have added some uncertainty to the results. The European Pet Food Industry Federation [56] recommends total fecal collection over a period of four days, which was not practically possible in our case. The difference in ATTD could have been expected to affect fecal DM, but no such difference was detected. However, we observed more sand and gravel in the feces following the R diet, and it is possible that the fecal DM content in R period samples would have been lower without this contamination.

Most dogs accepted all diets but the subjects were laboratory Beagle dogs, which are accustomed to eat what they are offered, and dogs of other breeds or privately owned dogs might be more selective. However, all dogs lost weight during all periods, which can be attributed to a number of factors. Apart from differences in ATTD, we calculated the daily energy requirement of the dogs based on the National Research Council recommendation for the average laboratory kennel dog, without considering individual variation. In addition, the outdoor temperature differed between periods and might have affected energy expenditure to a considerable extent. That was the reason for the increment in the daily ration in the RW diet period, the coldest period. Since some adjustments were made to the daily portion size and the weather conditions were different, no statistical analysis was performed on the weight loss.

Fecal samples are often used in studies examining gut microbiota composition and function, due to the non-invasiveness and ease with which they can be collected. We were interested in the effects of different cereal carbohydrate sources on microbial composition and production of SCFA and ATTD, and we assumed that fecal samples would give a fair approximation of these effects. One limitation is that samples taken from different parts of the intestine often differ in microbial composition [57]. However, the differences between colon and rectum have been shown to be non-significant [58], which indicates that fecal samples give a good reflection of the colonic microbiota. The composition and amount of SCFA have also been shown to shift in samples collected along the large intestine of pigs [42], presumably due to colonic uptake of SCFA and depletion of fermentable substrate. To our knowledge, no study has compared SCFA content in cecum/proximal colon fecal samples from dogs. However, given the considerably smaller cecum and shorter colon of dogs compared with pigs, these considerations might not be completely applicable to dogs. Therefore, fecal samples may give a reasonable approximation of SCFA produced along the colon.

Our study was limited by the small sample size. Large inter-individual variation was likely one reason why some of the differences between diets did not prove statistically significant. Further, although we did not observe any coprophagia, it might have occurred. This could have affected the microbiota, SCFA, and ATTD. To mitigate these effects, we did not apply a cross-over design in the experiments, even though some of the effects seen could then have been due to seasonal fluctuations in the microbiota. However, we did not see any differences in the baseline samples that could explain the differences after the diet periods, indicating that the changes were indeed due to diet.

## Conclusions

A diet with 50% rye inclusion (by DM) led to an increase in fecal relative abundance of the genus *Prevotella*, accompanied by a decrease in *Catenibacterium*, *Bacteroides*, *Romboutsia*, and *Megamonas.* This microbial shift seemed to affect the fiber fermentation pattern, as the relative proportion of acetic acid increased compared with other measured SCFA. ATTD was lower for the diet with 50% rye inclusion, compared with the other diets, indicating that a somewhat lower inclusion rate might be advisable. As *Prevotella* has been linked to stability in glucose metabolism and acetic acid has been observed to suppress appetite in humans, further studies on the effects of rye on metabolism and appetite in dogs are warranted.

## Supplementary Information

**Additional file 1:** **SupplementaryTable 1.** Compositionof WorkingDog Multivitamin (Trikem AB, Malmö, Sweden) in µg/mL, as stated bythe manufacturer. **SupplementaryTable 2.** Compositionof Calphosum D (Aptus, Orion Pharma Animal Health, Danderyd, Sweden) per gram, asstated by the manufacturer.

**Additional file 2:** **SupplementaryTable 3.** Dogbody weight in kg at end of the acclimatization period and at the end of eachdiet period. **SupplementaryTable 4.** Dog body condition score (BCS) at the end of theacclimatization period and at the end of each diet period.

## Data Availability

The datasets used and/or analyzed during the study are available from the corresponding author on reasonable request. The sequence data generated in this project has been deposited in the Sequence Read Archive (SRA): PRJNA816660.

## Abbreviations

ANOSIM: Analysis of similarity
ANOVA: Analysis of variance
ATTD: Apparent total tract digestibility
BCS: Body condition score
PCoA: Principal coordinate analysis
SCFA: Short-chain fatty acid
