# Supplementary material for: Effects of rye inclusion in dog food on fecal microbiota and short-chain fatty acids
Source: BMC Vet Res. 2023 May 10;19:70. doi: 10.1186/s12917-023-03623-2 (PMC10170736; doi:10.1186/s12917-023-03623-2)
Supplement: Supplementary file 1 — Additional file 1: SupplementaryTable 1. Compositionof WorkingDog Multivitamin (Trikem AB, Malmö, Sweden) in µg/mL, as stated bythe manufacturer. SupplementaryTable 2. Compositionof Calphosum D (Aptus, Orion Pharma Animal Health, Danderyd, Sweden) per gram, asstated by the manufacturer. [file 12917_2023_3623_MOESM1_ESM.docx]

**Additional file 1:**

**Supplementary table 1:** Composition of WorkingDog Multivitamin (Trikem AB, Malmö, Sweden) in µg/mL, as stated by the manufacturer

| Substance | µg/ml |
| --- | --- |
| Vitamin A | 54 |
| Vitamin D | 1 |
| Vitamin E | 2700 |
| Vitamin B1 | 27 |
| Vitamin B2 | 54 |
| Vitamin B6 | 27 |
| Vitamin B12 | 0.54 |
| Vitamin C | 2700 |
| Folic acid | 4 |
| Niacin | 54 |
| Pantothenic acid | 135 |
| Biotin | 5.4 |
| Copper | 27 |
| Iron | 440 |
| Manganese | 108 |
| Selenium | 2.7 |
| Choline | 2700 |
| Zinc | 490 |
| Iodine | 5.4 |
| Boron | 54 |

**Supplementary table 2:** Composition of Calphosum D (Aptus, Orion Pharma Animal Health, Danderyd, Sweden) per gram, as stated by the manufacturer

| Substance | unit/g |
| --- | --- |
| Calcium | 225 mg |
| Phosphorus | 115 mg |
| Vitamin D3 | 141 IE |
