# Supplementary material for: Effects of rye inclusion in dog food on fecal microbiota and short-chain fatty acids
Source: BMC Vet Res. 2023 May 10;19:70. doi: 10.1186/s12917-023-03623-2 (PMC10170736; doi:10.1186/s12917-023-03623-2)
Supplement: Supplementary file 2 — Additional file 2: SupplementaryTable 3. Dogbody weight in kg at end of the acclimatization period and at the end of eachdiet period. SupplementaryTable 4. Dog body condition score (BCS) at the end of theacclimatization period and at the end of each diet period. [file 12917_2023_3623_MOESM2_ESM.docx]

**Additional file 2:**

**Supplementary table 3:** Dog body weight in kg at end of the acclimatization period and at the end of each diet period

| Diet | Dog 1 | | Dog 2 | | Dog 3 | | Dog 4 | | Dog 5 | | Dog 6 | |
| --- | --- | --- | --- | --- | --- | --- | --- | --- | --- | --- | --- | --- |
|  | Start | End | Start | End | Start | End | Start | End | Start | End | Start | End |
| Wheat | 15.2 | 14.8 | 13.6 | 13.5 | 14.7 | 14.5 | 14.0 | 13.9 | 15.7 | 15.6 | 14.3 | 13.6 |
| Rye/Wheat | 15.0 | 14.2 | 14.0 | 13.5 | 14.9 | 14.7 | 14.6 | 14.0 | 15.6 | 15.1 | 13.8 | 12.9 |
| Rye | 15.6 | 15.3 | 13.7 | 13.3 | 15.0 | 14.4 | 14.8 | 14.6 | 16.3 | 16.0 | 14.1 | 13.2* |

*Weight at preterm exclusion

**Supplementary table 4:** Dog body condition score (BCS) at the end of the acclimatization period and at the end of each diet period

| Diet | Dog 1 | | Dog 2 | | Dog 3 | | Dog 4 | | Dog 5 | | Dog 6 | |
| --- | --- | --- | --- | --- | --- | --- | --- | --- | --- | --- | --- | --- |
|  | Start | End | Start | End | Start | End | Start | End | Start | End | Start | End |
| Wheat | 5 | 5 | 5 | 5 | 6 | 5 | 5 | 5 | 5 | 6 | 6 | 6 |
| Rye/Wheat | 5 | 4 | 5 | 5 | 6 | 6 | 6 | 6 | 6 | 5 | 6 | 5 |
| Rye | 5 | 4 | 5 | 5 | 5 | 5 | 6 | 6 | 6 | 5 | 5 | 5* |

*BCS at preterm exclusion
